# Enhancing Dementia Knowledge and Self-Efficacy of In-Home Supportive Services Caregivers Through Online Training

**DOI:** 10.1177/07334648221144023

**Published:** 2022-12-09

**Authors:** Jarmin Yeh, Brittney Pond, Matthew Beld, Andrea Garcia, Juvenal Mauricio, Juliana Mata-Pacheco, Corinne Eldridge, Leslie Ross

**Affiliations:** 1 Institute for Health & Aging, Department of Social & Behavioral Sciences, School of Nursing, 8785 University of California, San Francisco, CA, USA; 2 Center for Caregiver Advancement, Los Angeles, CA, USA

**Keywords:** Alzheimer's disease, caregiving, dementia, home care, training

## Abstract

California’s In-Home Supportive Services (IHSS) program provides vital home care to low-income consumers, some of whom live with Alzheimer’s disease or related dementias (ADRD). Yet, most IHSS caregivers receive little or no training in dementia care. We present preliminary descriptive results of the IHSS + ADRD Training Project, a 10-week, competency-based, dementia training program, delivered online, for IHSS caregivers, in Alameda County. Increase in dementia knowledge and self-efficacy at training completion supports the importance of continuing and expanding this work.

**What this paper adds**

• This paper adds to existing literature that new educational training models that use online and remote strategies can enhance IHSS caregivers’ dementia knowledge and skills to work with consumers living with ADRD.

**Applications of study findings**

• Our findings have implications for increased funding and training opportunities to bolster the roles and capacity of IHSS caregivers, especially as the prevalence of ADRD and the need for dementia-trained home care workers increases locally and statewide.

Successfully supporting people with Alzheimer’s disease and related dementias (ADRD) to live safely at home requires assistance from home care workers. In-Home Supportive Services (IHSS) caregivers are a type of home care worker directly hired by consumers of California’s Medi-Cal-funded IHSS program. Approximately, 550,000 IHSS caregivers provide custodial care and/or paramedical services to 650,000 IHSS consumers, the majority of whom are older adults (California Department of Social Services, n.d.). With ADRD prevalence among Californians age 55+ projected to increase 127% by 2040, reaching over 1.5 million people (Ross et al., 2021), the IHSS consumer population with ADRD will likely increase at a similar rate, amplifying the need for dementia-trained home care workers.

Since IHSS caregivers spend intimate time with consumers, they have the opportunity to observe changes in their consumer’s cognition, health, or behaviors to report to other family members or care team members. Caring for a consumer with ADRD is complex and can be physically and emotionally taxing. In-Home Supportive Services caregivers may be under-equipped to help consumers cope with environmental challenges (e.g., prevent wandering), manage comorbidities (e.g., diabetes), and may feel distress if/when expected to act as surrogate decision-makers (Parker et al., 2022; Sheehan et al., 2021). In-Home Supportive Services caregivers were essential workers during the Coronavirus Disease (COVID-19) pandemic (Espinoza, 2022), providing extended support during shelter-in-place, helping consumers prevent viral spread (e.g., masking and handwashing), and likely missing respite when their consumer would have been at an adult day program.

In-home supportive services caregivers have immense capacity to influence their consumer’s quality of care and reduce healthcare utilization (Newcomer et al., 2018). Without home care, many people with ADRD would have to live in costly institutions, such as nursing homes (Cook, 2017). Yet, IHSS caregivers remain an underutilized and undervalued resource in our long-term care system—most are hired family members (70%), they constitute a marginalized workforce composed of women (81%) and people of color (72%), receive minimal training or supportive supervision, and earn approximately US$16/h (California Department of Social Services, n.d.). Little is known about whether and how training could better support IHSS caregivers to thrive in their roles. Burgeoning research suggests that training programs are urgently needed, especially as the number of Californians with ADRD is projected to double by 2040 (Guerrero et al., 2020; Polacsek et al., 2020; Ross et al., 2021).

## Methods

The goal of the IHSS + ADRD Training Project is to implement and evaluate a 10-week dementia training program for 600 IHSS caregivers in Alameda County, California, by 2024. The curriculum contains 35-hours of content developed by the Center for Caregiver Advancement (CCA), a non-profit organization founded by home care workers (Guerrero et al., 2020). The project aims to increase IHSS caregivers’ dementia knowledge and self-efficacy to maximize care they provide to consumers.

Project funding and implementation began just prior to the onset of the COVID-19 pandemic. Consequently, the training launch was delayed by 6 months. In-person training pivoted to online training via Zoom, a video-conferencing platform, to comply with social distancing mandates.

This brief report presents preliminary results from the first cohort of IHSS caregivers who participated in the online training from September 2020 to March 2021. We used a quasi-experimental, longitudinal design.

### Participants

The Center for Caregiver Advancement recruited eligible IHSS caregivers through mailers, phone, text, internet, and social media outreach. Eligibility for IHSS caregivers included: age 18+, English-speaking, and hired by an IHSS consumer who is age 50+, has a score of 2+ on the Washington University Dementia Screening Test (AD8), and is a member of Alameda Alliance for Health (a local Medi-Cal managed care plan).

**Figure 1.**
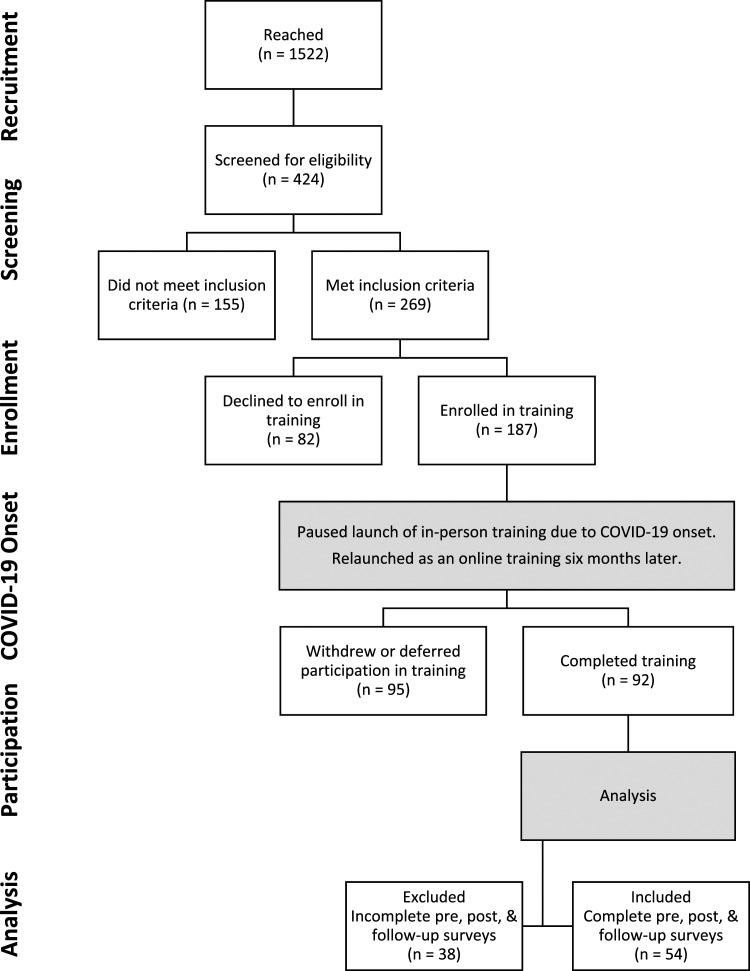
IHSS caregiver participant flow diagram.

Consent was obtained from IHSS caregivers and consumers or a designated power of attorney. Among 187 IHSS caregivers who initially enrolled, 95 withdrew or deferred participation and 92 completed the training when it relaunched as an online program 6 months later due to COVID-19 (Figure 1). Reasons for withdrawal or deferral included pandemic uncertainties; fraud, scam, or privacy concerns; technology barriers; illness; or the enrolled IHSS consumer died or no longer lived at home (e.g., placed in a facility), thereby disqualifying the caregiver.

### Data Collection

Data were collected using pre-training, post-training, and 3-month follow-up surveys. CCA distributed surveys to IHSS caregivers. Multiple choice questions included forced-choice responses and questions where multiple items could be selected. In-Home Supportive Services caregivers were also asked qualitative open-ended questions such as: “How do you plan on applying what you have learned from this training in your daily life?” To accommodate IHSS caregivers’ preferences and comfort level with digital literacy, paper mail-and-return and web-based Qualtrics surveys were available (Table 1). In-Home Supportive Services caregivers could receive up to $300 for completing the training and all surveys. Dementia knowledge, self-efficacy, distress, depression, and training satisfaction were primary caregiver outcomes.

**Table 1.** Overall Number and Percentage of Mail-and-Return Versus Qualtrics Surveys Received.

	Pre-training		Post-training	Follow-up	
	Part 1	Part 2		Part 1	Part 2
Paper	96 (89.7%)	96 (90.6%)	9 (10.7%)	9 (8.0%)	7 (6%)
Qualtrics	11 (10.3%)	10 (9.4%)	75 (89.3%)	103 (92%)	109 (94%)
Total	107	106	84	112	116

### Measures

The Washington University Dementia Screening Test (AD8) is a valid screening measure that differentiates between signs of normal cognition and mild dementia, which is completed by an informant (e.g., caregiver), the preferred administration method. It includes eight statements with response options of Yes/No/Don’t Know or N/A. Two or more “Yes” responses indicate cognitive impairment is likely present. The AD8 has a reliability coefficient of α = 0.84 (Galvin et al., 2006).

The Dementia Knowledge Assessment Tool 2 (DKAT2) is a valid self-assessment of dementia knowledge and includes 21 statements, with response options of Yes/No/Don’t Know. The more statements correctly answered indicates better dementia knowledge. The DKAT2 has a reliability coefficient of α = 0.79 (Toye et al., 2014).

The Caregiver Self-Efficacy Scale includes 10 statements, with a 1–10 rating scale, clustered into two domains: “Symptom Management” (5 statements; 5–50 score range) and “Community Support Service Use” (4 statements; 4–40 score range). One statement—“find ways to pay for services”—did not load into either domain. The domains have reliability coefficients of α = 0.77 and α = 0.78, respectively (Fortinsky et al., 2002).

The Caregiver Self-Assessment Questionnaire (CSAQ) is a valid self-report screening measure sensitive for detecting distress and includes 18 statements. The first 16 statements have Yes/No response options followed by two statements with a 1–10 rating scale. Chances of experiencing high distress are true if any of these criteria are met—(a) “Yes” is answered to either or both statements #4 and #11, (b) total “Yes” response is 10 or more, or (c) statements #17 or #18 score 6 or higher. The CSAQ has a reliability coefficient of α = 0.82 (Epstein-Lubow et al., 2010).

The Patient Health Questionnaire-2 (PHQ-2) is a valid self-report depression screener and includes two questions, with a 0–3 rating scale (Kroenke et al., 2003). The possible score range is 0–6, and a score 3+ has sensitivity for depressed mood over the past 2 week. The PHQ-2 had a reliability coefficient of α = 0.76 in an earlier study (Maroufizadeh et al., 2019).

### Analysis

Quantitative data were analyzed in Stata/SE 17. To compare demographics between the groups included and excluded for analysis, we performed chi-square tests. To compare changes in dementia knowledge and self-efficacy, we performed repeated measures univariate Analysis of Variance (ANOVA), with post-hoc comparisons adjusted by the Bonferroni test. An alpha value of 0.05 was used to consider differences statistically significant. Demographics, distress, depression, and training satisfaction were summarized with descriptive statistics.

## Results

Among 92 IHSS caregivers who completed the training, 54 were included for analysis and 38 were excluded due to incomplete surveys. Differences between included and excluded caregivers were significant by marital status, gender, and having people in their life they can ask for help. Table 2 displays IHSS caregiver demographics.

**Table 2.** IHSS Caregiver Characteristics.

Demographic descriptions	Total completed *n* = 92 (%)	Excluded *n =* 38 (%)	Included *n =* 54 (%)	*p*-value
Age, in years (SD)	69.2 (±11.8)	68.7 (±11.2)	69.6 (±12.7)	.74
Ethnicity				
Hispanic or Latinx	13 (14.1%)	5 (13.2%)	8 (14.8%)	.35
Not Hispanic or Latinx	60 (65.2%)	22 (57.9%)	38 (70.4%)	
Prefer not to answer/did not answer	19 (20.7%)	11 (28.9%)	8 (14.8%)	
Race^a^				
American Indian or Alaska native	5 (5.4%)	2 (5.3%)	3 (5.6%)	
Asian or Asian American	11 (12.0%)	1 (2.6%)	10 (18.5%)	
Black or African American	53 (57.6%)	27 (71.1%)	26 (48.1%)	
Native Hawaiian or other pacific islander	2 (2.2%)	—	2 (3.7%)	
White or Caucasian	8 (8.7%)	2 (5.3%)	6 (11.1%)	
Not listed	15 (16.3%)	9 (23.7%)	6 (11.1%)	
Prefer not to answer/did not answer	5 (5.4%)	3 (7.9%)	2 (3.7%)	
Marital status				
Married/partnered	38 (41.3%)	10 (26.3%)	28 (51.9%)	.01
Divorced	15 (16.3%)	10 (26.3%)	5 (9.3%)	
Separated	3 (3.3%)	3 (7.9%)	—	
Widowed	4 (4.3%)	1 (2.6%)	3 (5.6%)	
Never married	25 (27.2%)	8 (21.1%)	17 (31.5%)	
Prefer not to answer/did not answer	7 (7.6%)	6 (15.8%)	1 (1.9%)	
Gender identity				
Female	73 (79.3%)	23 (60.5%)	50 (92.6%)	.01
Male	17 (18.5%)	13 (34.2%)	4 (7.4%)	
Prefer not to answer/did not answer	2 (2.2%)	2 (5.3%)	—	
Sexual orientation				
Straight/heterosexual	78 (84.8%)	31 (81.6%)	47 (87.0%)	.65
Gay/lesbian/bisexual	3 (3.3%)	2 (5.3%)	1 (1.9%)	
Prefer not to answer/did not answer	11 (12.0%)	5 (13.2%)	6 (11.1%)	
Education				
K-12/GED	18 (19.6%)	8 (21.1%)	10 (18.5%)	.76
Some college—bachelors	68 (73.9%)	26 (68.4%)	42 (77.8%)	
Advanced degree	3 (3.3%)	2 (5.3%)	1 (1.9%)	
Prefer not to answer/did not answer	3 (5.5%)	2 (5.3%)	1 (1.9%)	
Household size				
Mean (*SD*)	3.4 (±1.7)	3.6 (±1.7)	3.1 (±1.5)	.19
1–3 people	51 (55.4%)	26 (68.4%)	25 (46.3%)	.07
4–9+ people	35 (38.0%)	10 (26.3%)	25 (46.3%)	
Prefer not to answer/did not answer	6 (6.5%)	2 (5.3%)	4 (7.4%)	
Annual household income				
US$0 to US$29,999	25 (27.2%)	11 (28.9%)	14 (25.9%)	.24
US$30,000 to US$79,999	34 (37.0%)	13 (34.2%)	21 (38.9%)	
US$80,000 to US$100,999	5 (5.4%)	—	5 (9.3%)	
Prefer not to answer/did not answer	28 (30.4%)	14 (36.8)	14 (25.9%)	
Years of IHSS experience				
Less than 1 year	1 (1.1%)	—	1 (1.9%)	.33
1–3 years	19 (20.7%)	10 (27.0%)	9 (16.7%)	
3–5 years	16 (17.4%)	7 (18.9%)	9 (16.7%)	
5–10 years	30 (32.6%)	8 (21.6%)	22 (40.7%)	
More than 10 years	24 (26.1%)	11 (29.7%)	13 (24.1%)	
Prefer not to answer/did not answer	2 (2.2%)	2 (5.4%)	—	
IHSS work hours/week				
Less than 10 hours	3 (3.3%)	1 (2.7%)	2 (3.7%)	.92
10–25 hours	26 (28.3%)	12 (32.4%)	14 (25.9%)	
25–40 hours	26 (28.3%)	10 (27.0%)	16 (29.6%)	
40–66 hours	25 (27.2%)	10 (27.0%)	15 (27.8%)	
66–90 hours	10 (10.9%)	3 (8.1%)	7 (13.0%)	
Prefer not to answer/did not answer	2 (2.2%)	2 (5.4%)	—	
Total IHSS consumers				
1	63 (68.5%)	29 (78.4%)	34 (63.0%)	.13
2	20 (21.7%)	5 (13.5%)	15 (27.8%)	
3	6 (6.5%)	1 (2.7%)	5 (9.3%)	
4	1 (1.1%)	1 (2.7%)	—	
Prefer not to answer/did not answer	2 (2.2%)	2 (5.4%)	—	
Job satisfaction				
Very dissatisfied	11 (12.0%)	4 (10.8%)	7 (13.0%)	.56
Dissatisfied	0 (0%)	0 (.0%)	0 (0%)	
Neither	3 (3.3%)	2 (5.4%)	1 (1.9%)	
Satisfied	36 (39.1%)	17 (45.9%)	19 (35.2%)	
Very satisfied	40 (43.5%)	14 (37.8%)	26 (48.1%)	
Prefer not to answer/did not answer	2 (2.2%)	1 (2.7%)	1 (1.9%)	
I feel I have the skills to do my job				
Strongly disagree	2 (2.2%)	2 (5.4%)	—	.06
Disagree	4 (4.3%)	3 (8.1%)	1 (1.9%)	
Neutral	11 (12.0%)	4 (10.8%)	7 (13.0%)	
Agree	45 (48.9%)	21 (56.8%)	24 (44.4%)	
Strongly agree	29 (31.5%)	7 (18.9%)	22 (40.7%)	
Prefer not to answer/did not answer	1 (1.1%)	1 (2.7%)	—	
I know I can meet all the needs of my consumer				
Strongly disagree	2 (2.2%)	2 (5.4%)	—	.18
Disagree	3 (3.3%)	2 (5.4%)	1 (1.9%)	
Neutral	13 (14.1%)	7 (18.9%)	6 (11.1%)	
Agree	33 (35.9%)	14 (37.8%)	19 (35.2%)	
Strongly agree	39 (42.4%)	12 (32.4%)	27 (50.0%)	
Prefer not to answer/did not answer	2 (2.2%)	1 (2.7%)	1 (1.9%)	
I have people in my life I can ask for help				
Strongly disagree	2 (2.2%)	2 (5.4%)	0 (0%)	.01
Disagree	1 (1.1%)	1 (2.7%)	0 (0%)	
Neutral	8 (8.7%)	4 (10.8%)	4 (7.4%)	
Agree	40 (43.5%)	22 (59.5%)	18 (33.3%)	
Strongly agree	39 (42.4%)	8 (21.6%)	31 (57.4%)	
Did not answer	2 (2.2%)	1 (2.7%)	1 (1.9%)	
Relationship to consumer				
Spouse/partner	6 (6.5%)	2 (5.4%)	4 (7.4%)	.31
Offspring	36 (39.1%)	12 (32.4%)	24 (44.4%)	
Offspring-in-law	2 (2.2%)	—	2 (3.7%)	
Another relative	5 (6.5%)	3 (8.1%)	3 (5.6%)	
Friend of neighbor	17 (18.5%)	9 (24.3%)	8 (14.8%)	
Homecare hired worker	8 (8.7%)	1 (2.7%)	7 (13.0%)	
Other	13 (14.1%)	7 (18.9%)	6 (11.1%)	
Did not answer	4 (4.3%)	4 (10.8%)	—	
Live in same household as consumer				
No	45 (48.9%)	17 (45.9%)	28 (51.9%)	.76
Yes	43 (46.7%)	17 (45.9%)	26 (48.1%)	
Did not answer	4 (4.3%)	4 (10.8%)	—	

^a^Participants had the option to select more than one category for race.

Among the 54 IHSS caregivers included for analysis, 70% were not Hispanic/Latinx, 48% Black/African American, 52% married/partnered, 93% female, and 87% straight/heterosexual. Average age was 69 (±12.7). Most had 5–10 years of IHSS experience (41%), worked for IHSS 25–40 hours/week (30%), cared for one IHSS consumer (63%), were very satisfied with their job (48%), agreed they have skills to do their job (44%), strongly agreed they know how to meet their consumer’s needs (50%) and have people in their life they can ask for help (57%). Most were the offspring of (44%) and did not live in the same household as their consumer enrolled in this program (52%).

### Knowledge

On average, IHSS caregivers correctly answered 11 of 21 statements at pre-training, compared to 15 at post-training and follow-up (Table 3). DKAT2 scores significantly increased from pre-training to post-training, and from pre-training to follow-up, but not from post-training to follow-up (Tables 4 and 5).

**Table 3.** Mean and Standard Deviation of the Dementia Knowledge Assessment Tool (DKAT2) and Caregiver Self-Efficacy Scale Scores.

Measures	Pre-training		Post-training		Follow-up	
	Mean	*SD*	Mean	*SD*	Mean	*SD*
DKAT2	11.9	3.6	15.9	2.2	15.7	2.1
Caregiver self-efficacy scale						
Symptom management domain	38.7	9.7	45.8	4.6	46.8	3.3
Community support service use domain	30.1	7.6	34.8	5.1	35.4	5.3

*Note.* DKAT2 possible score range is 0–21.

Symptom Management self-efficacy domain possible score range 5–50.

Community Support Service Use self-efficacy domain possible score range 4–40.

**Table 4.** Repeated Measure Univariate Analysis of Variance (ANOVA) Results of the Dementia Knowledge Assessment Tool (DKAT2) Scores.

Source	Unadjusted DF	Unadjusted mean square	F value	Unadjusted *p*-value	Adjusted *p*-value by G-G	Adjusted *p*-value by H-F
Survey	2	278.97	73.43	.00	.00	.00
Error (survey)	53	14.73				

Abbreviation: DF, Degrees of freedom; G–G, Greenhouse–Geisser epsilon; H–F, Huynh–Feldt epsilon.

**Table 5.** Pairwise Comparisons of Marginal Linear Predictions of the Dementia Knowledge Assessment Tool (DKAT2) Scores.

Survey	Contrast	Std. err.	Bonferroni		Bonferroni	
			t	*p*-value	95% CI	
Post-training versus pre-training	4	.38	10.66	.00	3.08	4.91
Follow-up versus pre-training	3.87	.38	10.32	.00	2.96	4.78
Follow-up versus post-training	−.13	.38	−.35	1.00	−1.04	.78

### Self-Efficacy

The mean “Symptom Management” domain score was 38.7 out of 50 at pre-training, compared to 45.8 at post-training and 46.8 at follow-up; the mean “Community Support Services Use” domain score was 30.1 out of 40 at pre-training, compared to 34.8 at post-training and 34.5 at follow-up (Table 3). Both “Symptom Management” and “Community Support Services Use” scores significantly increased from pre-training to post-training, and from pre-training to follow-up, but not from post-training to follow-up (Tables 6–9).

**Table 6.** Repeated Measure Univariate Analysis of Variance (ANOVA) Results of the Caregiver Self-Efficacy Scale Symptom Management Domain Scores.

Source	Unadjusted DF	Unadjusted mean square	F value	Unadjusted *p*-value	Adjusted *p*-value by G-G	Adjusted *p*-value by H-F
Survey	2	1048.13	29.28	.00	.00	.00
Error (survey)	53	55.25				

Abbreviation: DF, Degrees of freedom; G–G, Greenhouse–Geisser epsilon; H–F, Huynh–Feldt epsilon.

**Table 7.** Pairwise Comparisons of Marginal Linear Predictions of the Caregiver Self-Efficacy Scale Symptom Management Domain Scores.

Survey	Contrast	Std. err.	Bonferroni		Bonferroni	
			t	*p* value	95% CI	
Post-training versus pre-training	8.07	1.15	7.01	.00	5.27	10.88
Follow-up versus pre-training	7.09	1.15	6.16	.00	4.29	9.89
Follow-up versus post-training	−.98	1.15	−.85	1.00	−3.78	1.82

**Table 8.** Repeated Measure Univariate Analysis of Variance (ANOVA) Results of the Caregiver Self-Efficacy Scale Community Support Service Use Domain Scores.

Source	Unadjusted DF	Unadjusted mean square	F value	Unadjusted *p*-value	Adjusted *p*-value by G-G	Adjusted *p*-value by H-F
Survey	2	454.23	16.78	.00	.00	.00
Error (survey)	53	59.00				

Abbreviation: DF, Degrees of freedom; G–G, Greenhouse–Geisser epsilon; H–F, Huynh–Feldt epsilon.

**Table 9.** Pairwise Comparisons of Marginal Linear Predictions of the Caregiver Self-Efficacy Scale Community Support Service Use Domain Scores.

Survey	Contrast	Std. err.	Bonferroni		Bonferroni	
			t	*p* value	95% CI	
Post -training versus pre-training	5.28	1.00	5.27	.00	2.84	7.71
Follow-up versus pre-training	4.74	1.01	4.70	.00	2.29	7.20
Follow-up versus post-training	−.54	1.01	−.53	1.00	−2.99	1.92

### Distress

Caregiver self-assessment questionnaire scores indicated that 46% of IHSS caregivers were experiencing high distress at pre-training, compared to 50% at post-training and 35% at follow-up (Table 10). Significance testing was not conducted.

**Table 10.** IHSS Caregivers Who Screened Positively for Distress on the Caregiver Self-Assessment Questionnaire (CSAQ) and Depression on the Patient Health Questionnaire-2 (PHQ-2), *n* = 54.

Measure	Pre-training	Post-training	Follow-up
CSAQ (high distress)	25 (46.3%)	27 (50%)	19 (35.2%)
PHQ-2 (depressed mood)	4 (7.4%)	6 (11%)	3 (5.6%)

*Note.* Significance testing was not conducted.

### Depression

PHQ-2 scores indicated that 7% of IHSS caregivers were experiencing depressed mood at pre-training, compared to 11% at post-training and 6% at follow-up (Table 10). Significance testing was not conducted.

### Satisfaction

At post-training, 94% of IHSS caregivers were very satisfied with the training. In-Home Supportive Services caregivers strongly agreed the training was beneficial (90%), they learned new caregiving skills (82%), communication with their consumer improved (72%), the instructor made them feel comfortable (91%) and effectively answered questions (91%) (Table 11). They shared how they would apply learnings to their daily life in open-ended questions. For instance, one IHSS caregiver explained: “I plan to speak clearly, repeat willingly, offer choices whenever possible. Keep records (documentation) and learn as much as possible about the consumer before their present condition.”

**Table 11.** IHSS Caregivers’ Training Satisfaction, *n* = 54.

	Agree	Strongly agree
Participating in this training was beneficial to me	3 (5.6%)	49 (90.1%)
I believe I have learned new caregiving skills because of this training	8 (14.8%)	44 (81.5%)
Communication with my IHSS consumer has improved because of this training	12 (22.2%)	39 (72.2%)
The instructor made me feel comfortable	4 (7.4%)	49 (90.7%)
The instructor was able to answer questions effectively	4 (7.4%)	49 (90.7%)

	Satisfied	Very satisfied
Overall, how satisfied were you with this training?	2 (3.7%)	51 (94.4%)

## Discussion

Our preliminary results revealed promising signs that online training can bolster the capacity of IHSS caregivers to better support their consumers living with ADRD or cognitive impairment. Dementia knowledge and self-efficacy significantly increased at post-training, with trends suggesting retention at 3-month follow-up. These results are consistent with positive outcomes found in the growing research on online dementia training efforts for caregivers (Pleasant et al., 2020), and supports the importance of continuing and expanding this work with home care workers (Guerrero et al., 2020; Polacsek et al., 2020).

This study had several limitations. Those who opt-in for eligibility screening may be more aware of ADRD and cognition changes in their consumer, which is potential selection bias. Switching to online and remote strategies introduced new processes and adaptations. Tracking and receiving complete data from IHSS caregivers through mail-and-return surveys was resource-intensive and not always reliable. Some IHSS caregivers were ambivalent about sharing personal information online due to heightened fears of fraud and scams. We did begin receiving more Qualtrics surveys at post-training and follow-up, suggesting growing comfort with using Qualtrics (Table 1). Those who responded to surveys may have also been influenced by selection bias. It was difficult to interpret if distress and depression measures captured or reflected fluctuating effects from the COVID-19 pandemic occurring concurrently. Self-reported data by IHSS caregivers were subject to recall and desirability biases. Finally, this study had no control group comparison.

Future analyses will include multivariate regression models to establish relationships between sociodemographic variables and caregiver outcomes, and compare healthcare utilization patterns of IHSS consumers before and after their caregiver’s participation in the training. Additional cohorts with classes in English, Spanish, and Cantonese are already underway. This work has implications for expansion to other California counties beyond Alameda as prevalence of ADRD and demand for dementia-trained home care workers rise statewide.
